# Comparative Analysis of Main Agronomic Traits of Different *Pleurotus giganteus* Germplasm Resources

**DOI:** 10.3390/life14020238

**Published:** 2024-02-08

**Authors:** Miaomiao Yan, Dandan Zhai, Qiaozhen Li, Meiyan Zhang, Ning Jiang, Jianyu Liu, Chunyan Song, Xiaodong Shang, Hongyu Chen, Hailong Yu

**Affiliations:** 1 Institute of Edible Fungi, Shanghai Academy of Agricultural Sciences, National Engineering Research Center of Edible Fungi, Shanghai 201403, China; yanmiaomiao0331@163.com (M.Y.); zhaidandan0519@163.com (D.Z.); liqiaozhen@saas.sh.cn (Q.L.); zhangmeiyan@saas.sh.cn (M.Z.); jiangning@saas.sh.cn (N.J.); liujianyu@saas.sh.cn (J.L.); songchunyan@saas.sh.cn (C.S.); shangxiaodong@saas.sh.cn (X.S.); 2 Engineering Research Centre of Chinese Ministry of Education for Edible and Medicinal Fungi, Jilin Agricultural University, Changchun 130118, China

**Keywords:** *Pleurotus giganteus*, mycelium running, agronomic traits, biological efficiency

## Abstract

Agronomic traits are key components in variety protection, cultivar development, and the formulation of DUS (distinct, uniform, and stable) test guidelines. *P. giganteus* is an increasingly popular and commercially promising edible macrofungi. In this study, both mycelial performance and fruiting body characters of 15 *Pleurotus giganteus* strains were investigated. The temperature gradient culture test indicated that, although most of the strains achieved optimal mycelial growth between 24 and 28 °C, a statistical difference in mycelial growth rates and temperature adaptability among strains were found, supporting that this trait has the potential to be adopted as an indicator in distinguishing strains. In the fruiting performance tests, the coefficient of variation (CV) of tested traits ranged from 5.30% (pileus diameter) to 18.70% (individual mushroom weight). The mushroom yields ranged from 103.37 g/bag (strain No. 15) to 275.76 g/bag (strain No. 9). The large divergence observed in individual mushroom weight tested strains, ranging from 40.88 g to 78.39 g (with median between 37.69 and 79.395 g), make it highly selective and a potential indicator in variety development. Strain No. 9 had the advantages of forming larger, heavier fruiting bodies and a more obvious funnel shape, which also exhibited the highest biological efficiency (15.61%). The results suggested some morphological traits showed high variety difference, such as pileus diameter (55.75 mm to 66.48 mm), stipe length (92.59 mm to 177.51 mm), stipe diameter (16.14 mm to 23.52 mm), and pileus thickness (13.38 mm to 19.75 mm). In the cluster analysis, the tested strains were grouped into four clusters based on agronomic traits: cluster Ⅰ comprised six strains (No. 6, No. 11, No. 8, No. 1, No. 14, and No. 9) with high mushroom yield; cluster Ⅱ included four strains (No. 3, No. 10, No. 7, and No. 4) with large pileus diameter and short stipe; cluster ⅡI consisted of four strains (No. 5, No. 12, No. 13, and No. 15) with relatively lower yields; and cluster Ⅳ included only strain No. 2 which was low in yield, individual mushroom weight, and biological efficiency, accompanied by smaller pileus size and shorter stipe. The results of the correlation analysis indicated three traits, including individual mushroom weight, stipe length, and pileus weight, were positively associated with high yield. This study suggested *P. giganteus* germplasm resources are of high abundance and their agronomic diversity is useful in distinguishing and developing different varieties. The findings of this work provide knowledge on the agronomic traits and cultivation performance of various *P. giganteus* strains, laying a foundation for the development of its DUS test guidelines and variety protection, as well as providing reference for the breeding and phenotype selection of high-quality cultivars.

## 1. Introduction

*Pleurotus giganteus*, also known as giant oyster mushroom or giant funnel mushroom, is a nutritious edible mushroom taxonomically categorized into Basidiomycota, Agaricomycetes, Agaricales, Pleurotaceae [1]. *P. giganteus* has a unique flavor and delicious taste. It is rich in nutrients, especially protein, whose essential amino acids contribute to 43% of the total amino acid pool, higher than *Lentinula edodes* and *P. ostreatus* [2,3]. Other nutrients and bioactive phytochemicals such as carbohydrates, minerals, triterpenoids [4], and polysaccharides [5] are also found in abundance in *P. giganteus*. The bioactive compounds isolated from the fruiting body and mycelium of *P. giganteus* have been reported to have various health benefits such as immunity augmentation, anti-oxidant activities [4], anti-fungal effects [6], liver protection [7], anti-inflammation [8], anti-diabetes effects [9], and anti-tumor effects [10], showing broad potential medicinal value.

The first cultivar was isolated and domesticated from a wild strain from the Sanming Institute of Fungi in Fujian, China in the 1980s. To date, nearly 250 strains of *P. giganteus* have been found and recorded in different regions of the world, such as China, Malaysia, Sri Lanka, Indonesia, Vietnam, Laos, and Thailand. The current germplasm resources used for *P. giganteus* production are mainly obtained from the domestication or tissue isolation of wild resources. *P. giganteus* is a high-value edible mushroom that is easy to cultivate and commercialize. Its production advantages such as strong growth adaptability, wide range of substrate options, high temperature tolerance, high biological efficiency, and long shelf life allow it to grow in warm seasons and expand to tropical regions. Currently, the main producing areas in China include Guangdong, Fujian, Zhejiang, Shandong, and Beijing. Three modes for *P. giganteus* production are typically applied which include the following: (1) convenient cultivation incorporating seasonal farming based on climate conditions or off-season production using basic greenhouse facilities; (2) factory cultivation which provides a completely controlled environment within hermetic chambers and allows annual production; and (3) understory production which mainly relies on natural climate.

In recent years, the edible mushroom industry has been continuously developing and scaling-up, leading to an increasing demand for new varieties [11]. However, due to the late start of research on *P. giganteus* and a lack of research capacities, increasing challenges have emerged such as unclear germplasm resources and a lack of DUS (distinctness, uniformity, and stability) test guidelines for variety evaluation [12]. In order to motivate continuous variety improvement, conducting trait investigations on existing germplasm resources, exploring useful strains, and establishing evaluation systems are crucial tasks [13,14].

Testing of agronomic traits of fungal germplasms includes both mycelial performance and fruiting body characters. In this study, 15 *P. giganteus* strains were analyzed and compared for agronomic diversity. Mycelial growth rates were measured at different temperatures between 20 and 30 °C to test strain robustness and temperature adaptability. The strains were cultivated following established protocol for production [15]. Morphology and yield of fruiting bodies were analyzed to compare varieties and evaluate quality. Biological efficiency was calculated to investigate substrate utilization and *P. giganteus* growth. A correlation analysis and a cluster analysis of agronomic traits were carried out to determine the representativeness and divergence of the tested germplasm resources. The findings of this study provide data for the formulation of DUS test guidelines for *P. giganteus* and provide reference for the collection and breeding of high-quality varieties.

## 2. Materials and Methods

### 2.1. Test Materials

Fifteen strains were collected from different provinces in China (Table 1) and tested for mycelial performance as well as agronomic traits.

### 2.2. Medium

PDA medium for mother culture (g/L): 200 g of potato, 20 g of glucose, 20 g of agar, natural pH, mixed in 1 L of water.

Substrate for cultivation: 39% mixed sawdust, 39% cottonseed hull, 20% bran, 2% calcium carbonate, stirred with water to 53~55% moisture content, natural pH.

### 2.3. Determination of Mycelial Growth Rate

A 5 mm mycelium disc was inoculated on the center of a 90 mm petri dish containing PDA medium and then cultured in at 20 °C, 22 °C, 24 °C, 26 °C, 28 °C, and 30 °C, respectively, avoiding light. The growth rate of mycelium was measured by the cross-marking method. The diameter of the colony was recorded from the 3rd day after inoculation, and at the end of culture. The average daily growth rate of mycelia was recorded every 3 days and calculated. Each strain was inoculated and tested on 5 petri dishes.

### 2.4. Fruiting Body Cultivation

According to the formula of the cultivation material in Section 2.2, the substrate materials were mixed evenly in proportion and the water content was controlled between 53 and 55%. The prepared substrates were filled into a polypropylene bag with dimensions 15 × 20 cm (1 kg per bag). The substrate bags were autoclaved at 121 °C for 4.5 h, cooled to room temperature, and then inoculated with spawn at a ratio of 3%. The inoculated bags were transferred to multi-layer shelves in a culture chamber with a temperature of 22–23 °C, relative humidity of 70–80%, and CO_2_ concentration < 2000 ppm, avoiding light. The spawn run was carried out for 30 days until the mycelia grew and reached the bottom of the bag. Another 30 days of culture in the same condition was continued to allow mycelia to mature. After maturation of the mycelia (i.e., a total of 60 days since inoculation), the bags were opened and the room temperature was increased to 27 °C for primordia induction and fruiting body production. Then, the biomass were covered with soil and managed following Niu et al.’s [16] method to form fruiting bodies. Each strain was cultivated with 60 bags. The fruiting bodies were harvested for morphological observation and agronomic trait testing.

### 2.5. Test of Agronomic Characters of Fruiting Bodies

**Figure 1:**
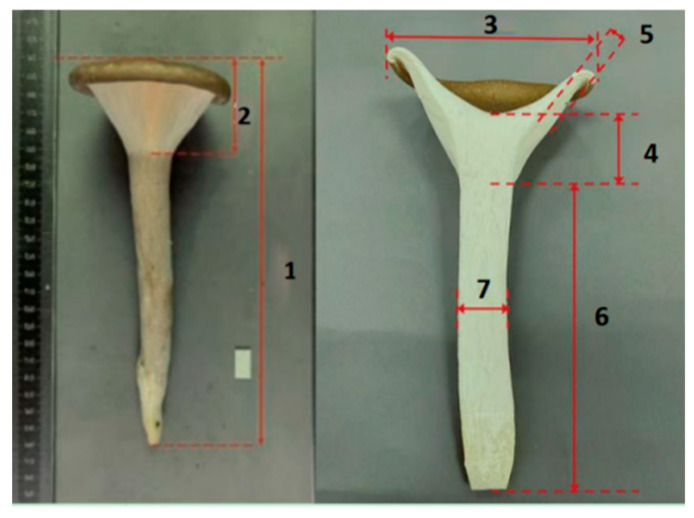
Definition and test method of each part of *Pleurotus giganteus* fruiting body. 1: Fruiting body; 2: pileus; 3: pileus diameter; 4: pileus thickness; 5: gill width; 6: stipe length; 7: stipe thickness.

The seven main agronomic traits of fruiting bodies and their testing methods are shown in Table 2 and Figure 1 [17,18].

### 2.6. Method for Determining Biological Efficiency

The biological efficiency of each strain was calculated as follows:

$$Biological\;efficiency\;(\%)=\frac{Weight\;of\;fresh\;mushroom\;(g)}{Weight\;of\;substrate\;before\;adding\;with\;water\;(g)}$$

### 2.7. Cluster Analysis

The agronomic traits of tested *P. giganteus* strains were analyzed by cluster mapping using SPSS 20.0 software (IBM Corp., Armonk, NY, USA) based on Euclidean distance, and a dendrogram was constructed [19].

### 2.8. Data Processing

Data were expressed as mean ± SD, where mycelial growth data were obtained from 5 petri dishes, and fruiting body agronomic traits data were obtained from 60 cultivation bags. Excel (Microsoft Corp., Redmond, WA, USA) was used to analyze the obtained data, SPSS 20.0 (IBM Corp., Armonk, NY, USA) was used for a one-way ANOVA and difference significance test (Duncan, *p* < 0.05), and OriginPro 2021 software (OriginLab Corp., Northampton, MA, USA) was used for mapping.

## 3. Results

### 3.1. Mycelial Growth Rate

**Figure 2:**
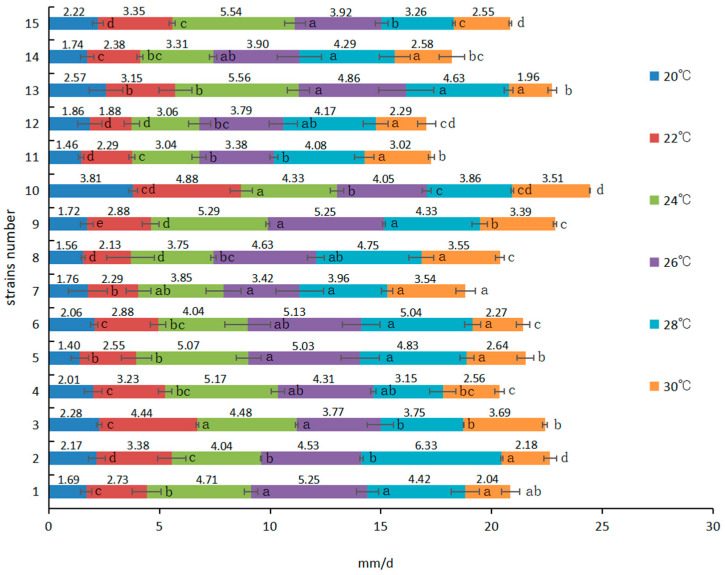
Mycelial growth rate of 15 strains at different temperatures. Different lowercase letters indicate significant difference at *p* < 0.05 level.

Fifteen strains of *P. giganteus* were cultured at different temperatures to determine their mycelial growth rates (Figure 2). Generally, the growth rates of each strain accelerated as the temperature rose until reaching its most suitable temperature and then decreased. Most of the strains grew faster between 24 and 28 °C, while statistical differences suggest diversity in growth rates and temperature adaptability. Two out of the fifteen tested strains (No. 1 and No. 6) showed the highest mycelial growth rate at 26 °C. Six strains (No. 2, No. 7, No. 8, No. 11, No. 12, and No. 14) achieved a maximum mycelial growth rate at 28 °C, whereas strain No. 2 (6.33 mm/d) grew much faster than strain No. 11 (4.08 mm/d). The mycelial growth rate of six strains (No. 3, No. 4, No. 5, No. 9, No. 13, and No. 15) reached a peak when the temperature was 24 °C. When strain No. 10 was cultured at 22 °C, its growth rate was maximized and higher than at other temperatures.

### 3.2. Agronomic Traits and Biological Efficiency

**Figure 3:**
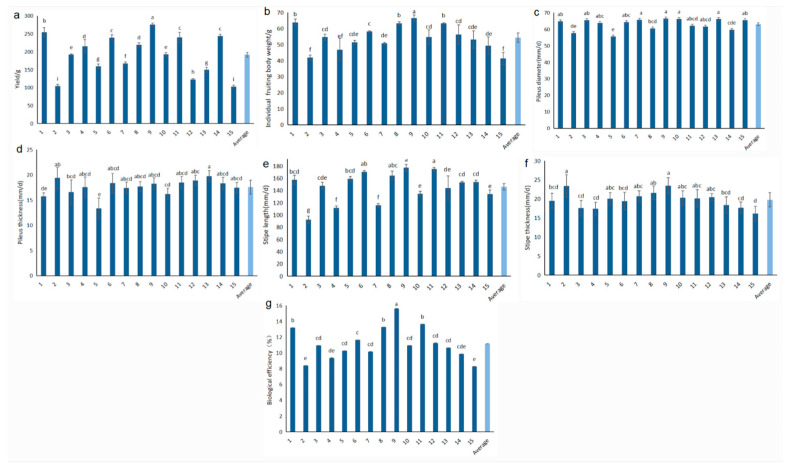
Agronomic characters of fruiting bodies and biological efficiency. (**a**): yield; (**b**): individual mushroom weight; (**c**): pileus diameter; (**d**): pileus thickness; (**e**): stipe length; (**f**): stipe diameter; (**g**): biological efficiency. Different lowercase letters indicate significant difference at *p* < 0.05 level.

The yields and morphological features exhibited large variance among the 15 tested strains (Figure 3). Among them, the yield of strain No. 9 was the highest, reaching 275.76 g per bag, with a significant difference as compared with other strains, while strain No. 15 had the lowest yield of only 103.37 g per bag (Figure 3a). Individual mushroom weight was observed with large divergence among tested strains, ranging from 78.39 g (reported from strain No. 9) to 40.88 g (reported from strain No. 15) (Figure 3b). The pileus diameters of strains No. 7, No. 9, No. 10, and No. 13 were larger in comparison to other strains (Figure 3c). The maximum average size of the pileus was reported in strain No. 9 (66.48 mm), while the minimum size was found in strain No. 5 (55.75 mm). The maximum pileus thickness was 19.75 mm (strain No. 13), yet the minimum pileus thickness was only 13.38 mm (strain No. 5) (Figure 3d). The longest stipe length was 177.51 mm (strain No. 9) and the shortest length was 92.59 mm (strain No. 2) (Figure 3e). The stipe diameter was as high as 23.52 mm for strain No. 9, yet as low as 16.14 mm for strain No. 15 (Figure 3f). The highest biological efficiency was obtained from strain No. 9 (15.61%), whereas the lowest efficiency, obtained from strain No. 15, was only 8.27% (Figure 3g). In summary, strain No. 9 showed the best performance in yield, strain No. 5 was characterized by small and thin pileus, and strain No. 11 produced long stipe and large-sized pileus, indicating the germplasm abundance and morphological diversity of *P. giganteus*.

Further analysis of the coefficient of variation (CV) of each trait revealed that the CVs of the seven agronomic traits of 15 strains ranged from 5.30% to as high as 18.70%. Among which, the highest CV was reported for individual mushroom weight, suggesting that the value of this indicator varied greatly among fruiting bodies. The pileus diameter was observed with the lowest CV, reflecting a relative uniformness of *P. giganteus* individuals for this trait.

### 3.3. Distribution Analysis of Individual Mushroom Weight

**Figure 4:**
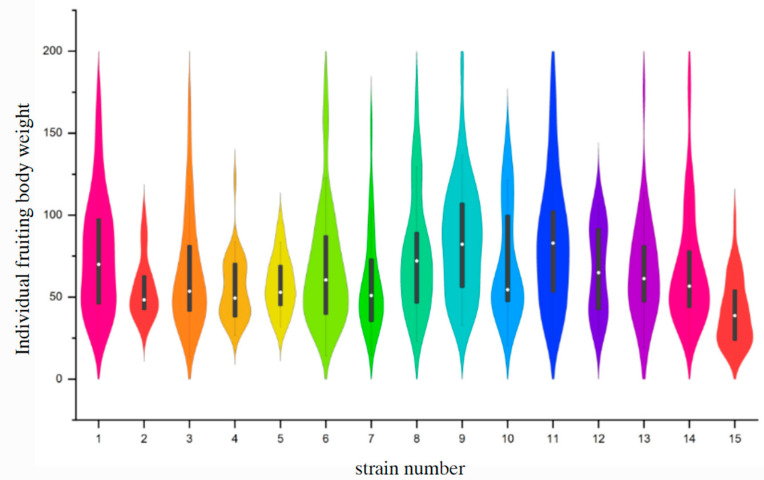
Violin plots of individual fruiting body weights (the violin plots depict distribution and kernel density of individual mushroom weights obtained from different strains. The thick grey bar represents the interquartile range, the white dot represents the median, and the top and bottom of each violin are the maximum and minimum mushroom weights, respectively).

According to the violin plots (Figure 4), the medians of individual fruiting body weight ranged from 37.69 g to 79.395 g. Strains No. 9 and No. 11 produced larger mushrooms (individual fruiting body weight > 180 g) rather than smaller mushrooms (minimum individual fruiting body weight > 18 g). The median (69.88 g) and upper quartile (106.22 g) of strain No. 9 were the largest, indicating its tendency to form bigger fruiting bodies. Strain No. 1 and No. 8 secondarily yielded relatively heavier fruiting bodies. The minimum median was obtained from strain No. 15, whose data were clustered in the lower quartile. There was a notable divergence among tested strains when navigating the median within the interquartile range, where strains No. 11 and No. 8 had a median closer to the maximum mushroom weight. Conversely, the median of strains No. 10, No. 2, No. 3, No. 4, and No. 5 were closer to the minimum mushroom weight. The distribution of data supported diversity among strains. Fruiting body weights of strains No. 5 and No. 13 were more clustered near the median, relatively in correspondence to the normal distribution. A symmetrical, elongated distribution was observed in strains No. 1, No. 9, and No. 11, indicating the numbers of mushrooms with different weights were similar. In addition, the lower quartiles were found wider in strains No. 2, No. 3, No. 4, No. 6, No. 10, No. 14, and No. 15, suggesting clustering and a more frequent occurrence of lighter individuals. Simultaneously, the elongated upper quartiles observed in strains No. 2, No. 3, No. 6, No. 7, No. 8, and No. 10 denoted a more dispersed distribution of heavier individuals. The fruiting body weight of strain No. 12, nevertheless, fluctuated greatly. Overall, strain No. 9 had an advantage in the production of larger mushrooms.

### 3.4. Cluster Analysis of Mushroom Agronomic Traits

**Figure 5:**
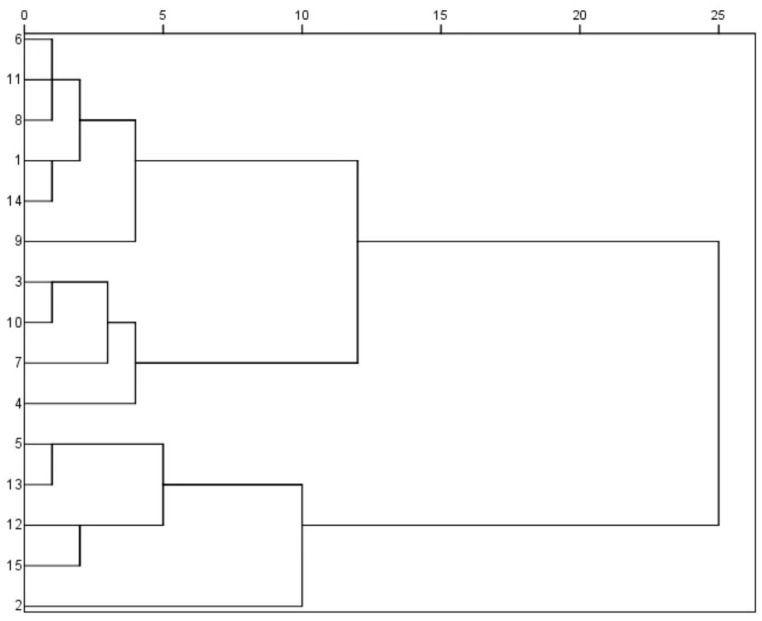
Dendrogram of cluster analysis of agronomic traits of *P. giganteus*.

A cluster analysis classified the 15 strains into five categories according to agronomic traits (Figure 5). The first cluster comprised strains No. 6, No. 11, No. 8, No. 1, No. 14, and No. 9, which were characterized by high mushroom yield, showing commercial advantages. The second cluster included strains No. 3, No. 10, No. 7, and No. 4, which exhibited features of large pileus diameter and short stipe. The third cluster included four strains with relatively lower yields. Strain No. 2 consisted of the fourth cluster which was low in yield, individual mushroom weight, and biological efficiency, accompanied by smaller pileus size and shorter stipe. After a comprehensive consideration of various agronomic traits, it was found that the agronomic traits of strain No. 2 and No. 9 were complementary, and the genetic relationship was distant.

### 3.5. Correlation Analysis of Mushroom Agronomic Traits

**Figure 6:**
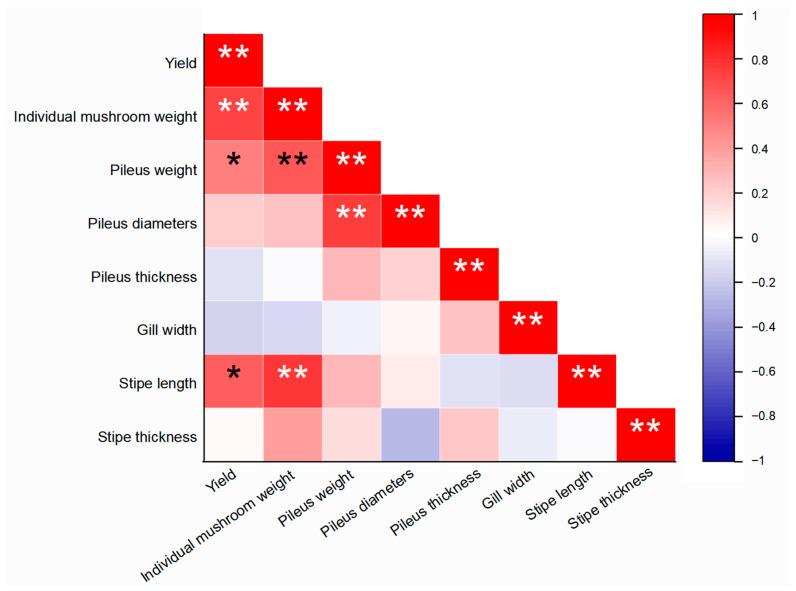
Correlation matrix of agronomic traits of *P. giganteus.* “*” and “**” represent significance at the 0.05 and 0.01 levels, respectively.

A correlation analysis between agronomic traits was conducted to combine important features and facilitate phenotype selection (Figure 6). The results suggested yield was significantly positively correlated with the individual mushroom weight (r = 0.731, *p* < 0.01), as well as pileus weight (r = 0.516, *p* < 0.05) and stipe length (r = 0.623, *p* < 0.05), whereas there was no significant correlation with other traits. The individual mushroom weight was positively correlated with pileus weight (r = 0.643, *p* < 0.01) and the stipe length (r = 0.768, *p* < 0.01). There was also a strong correlation between pileus weight and diameter (r = 0.745, *p* < 0.01). No significant correlation was observed between other traits. The results associated production indicators with some of the key morphological traits, providing further reference for *P. giganteus* variety protection and DUS test guideline formulation.

## 4. Discussion

Scientific evaluation of *P. giganteus* germplasm is a prerequisite for effective industrialization. A systematic identification and study of agronomic performance provides the basis and details for the breeding and protection of excellent varieties. The results of this work suggest *P. giganteus* germplasm resources are of high abundance, and their agronomic diversity is useful in distinguishing and developing different varieties. The morphological data provides database support for industrial practice and variety protection. In addition, breeders are also exploring cultivars with high yield and production adaptability [20].

Mycelial growth is an essential trait in studying edible fungi and a potential indicator in DUS testing to distinguish strains. Aiming to centrally test the mycelial growth performance, a standardized classical culture test was conducted in this study at a wide range of temperatures between 20 and 30 °C. Most of the strains achieved optimal mycelial growth between 24 and 28 °C. The results indicated a statistical difference in growth rates and temperature adaptability, supporting the use of mycelial growth rate at representative temperatures as an indicator in DUS testing to distinguish *P. giganteus* strains. Generally, the growth condition of *P. giganteus* is consistent with many industrialized edible macrofungi. Specifically, its optimal mycelial growth temperature is usually higher than that of *Lentinula edodes* and *Flammulina filiformis* [21]. In *P. giganteus* production practice, to ensure the successful development of fruiting bodies, an empirical technology is to control the chamber temperature during spawn run (22–23 °C) at slightly lower than optimal mycelial growth temperature (24–28 °C). The first reason is that, as a result of heat release from mycelial metabolism, the temperature inside the cultivation bag is typically 1–3 °C higher than the environmental temperature. In addition, the observed mycelium growth is more robust at slightly lower temperature which facilitates higher yield. Hence, the established cultivation protocol required 22–23 °C for spawn running [15]. In the current study, we followed standard protocol with environmental consistency to centrally test agronomic traits and preliminary screen strains. For future directions, more accurate temperature controls might be applied for the subsequent development of strain and cultivation techniques.

*P. giganteus* is an edible mushroom with great market potential. As its commercial value gradually gains widespread recognition and the cultivation scale continues to expand, more research is needed on genetic breeding, cultivation, and domestication of more varieties to speed up the development of excellent cultivars. At present, *P. giganteus* is mainly sold fresh and the market price is relatively high. Its size and quality of fruiting body are the main concerns of consumers and the major goals of breeders. *P. giganteus* typically grows solitarily, making individual mushroom weight an important indicator for growers. Especially, the CV of individual mushroom weight in this study was high, indicating this trait is highly selective in productional variety development. However, yield and mushroom size are not the only criteria in variety development. For *P. giganteus,* the shape of the pileus (funnel-shaped), the proportion of pileus weight, the timing of mushroom emergence, the necessity of covering soil during production, and other production and commercial indicators are all influential in industrial practice. In our tested strains, different strains showed divergent traits and production performance. For instance, strain No. 5 had the characteristics of a smaller pileus diameter and smaller thickness, while strain No. 11 had the features of a longer stipe length and larger pileus diameter, indicating the morphological abundance and diversity of fruiting bodies. Specifically, strain No. 9 had the advantages of forming larger fruiting bodies and a more obvious funnel shape, which is more preferred by consumers and suitable for the current market. Meanwhile, it is also characterized by high yield and individual fruiting body weight, hence, it is promising in equipped conditions and factory production. However, this strain showed the weakness of requiring approximately 7–10 days for fruiting bodies to appear after covering with soil, resulting in a long production cycle and high cost. Whereas strain No. 2 required a short time (4–5 days) to form primordium and produce mushrooms after covering with soil, and hence is recommended in understory production. The complementarity features make these strains auspicious candidates for hybridization parents. The goal of subsequent hybrid breeding on the basis of these findings is to further cut the mushroom fruiting time, together with obtaining favored traits, thereby shortening the production cycle, enhancing consumer acceptance, and increasing economic benefits [22,23].

As variety protection raises increasing interests among breeders and growers, investigations of agronomic traits and formulating DUS guidelines play a positive role in motivating continuous cultivar innovation and healthy development of the industry [14]. In this work, some representative traits showing high variety difference were selected, such as mycelial growth rate, pileus diameter, stipe diameter, and pileus thickness, which are subsequently expected to be used as indicators in DUS testing. One special point in the DUS test for edible mushrooms is that they are indeed more susceptible to environmental factors as compared to field crops. In DUS testing practice, it is recommended to minimize the cofounding impact on distinguishing varieties to require all test and reference varieties to be cultivated in the same condition to ensure environmental consistency, and to ensure enough cultivation scale and sufficient sample size to reduce individual differences, as well as to conduct centralized testing at designated premises to ensure experimental parallelism and data accuracy [13]. For edible mushrooms, it is not expected that all strains would exhibit differences in all test traits in one environment. In the current DUS testing practice in China, when a test variety shows significant and reproducible difference in at least one trait as compared with reference variety, it is determined with distinctness.

In addition, it should be noted that there is regional divergence in climate and environment. Although in DUS testing practice for edible fungi, environmental consistency and centralized testing at designated sites are required to minimize the impact of confounding factors on the test results and ensure experimental validity [13]. For producers and farmers, adjustment can be made in the used variety and cultivation facility management according to their own production mode and marketing demands, such as selecting a cultivar with temperature adaptability to local weather, and balancing budget, equipment, and substrate availability to choose an optimal cultivation method [20]. Particularly, convenient cultivation with lower cost requires strains and techniques favoring early fruiting. Factory production matches the case of batch production when fruiting timing of mushrooms is close, while understory production relying on natural conditions requires dispersed growth of mushrooms.

According to seven agronomic traits, the 15 strains were grouped into five clusters, indicating the richness of variety resources and the representativeness of selected strains. When tracing the origin of the strains, the majority of them came from south China, which may be related to the distribution of *P. giganteus* in southern China, such as Guangdong, Fujian, Zhejiang, and Yunnan. The correction analysis among morphology indicators and yield reported a positive association between some of the traits. Given the fact that *P. giganteus* has a highly fibrous stipe, the results highlighted that three traits including individual mushroom weight, stipe length, and pileus weight are recommended as the reference indexes for the selection and breeding of high-yield varieties.

For the present study, the differences in main traits of *P. giganteus* mycelium or fruiting body were analyzed. The findings shed light on the identification of germplasms and the formulation of DUS test guidelines, and provided a reference for the development of promising cultivars. Furthermore, to deepen our understanding of *P. giganteus* germplasm, it is expected to increase the collection of strains and investigate the genetic background in addition to morphological trait-based evaluation, and subsequently correlate the genotype data and phenotype data to identify the control genes of agronomic traits [24,25,26].

## Figures and Tables

**Table 1:** Fifteen strains were collected from different origins.

Number	Name	Source	
1	T212	Putian Agricultural Science Research Institute	Fujian, China
2	T26297	Putian Agricultural Science Research Institute	Fujian, China
3	T224	Putian Agricultural Science Research Institute	Fujian, China
4	ZD-FJ	Fujian Academy of Agricultural Sciences	Fujian, China
5	Tai an H	Tai’an Academy of Agricultural Sciences	Shandong, China
6	Pg_GX	Guangxi Academy of Agricultural Sciences	Guangxi, China
7	Pg_ZZZ	Fujian Agriculture and Forestry University	Fujian, China
8	Pg_FJB	Fujian Agriculture and Forestry University	Fujian, China
9	SX1	Shanghai Academy of Agricultural Sciences	Shanghai, China
10	Pg_LD	Ludong University	Shandong, China
11	Pg_GXB	Guangxi Academy of Agricultural Sciences	Guangxi, China
12	Pg_JL1	Jilin Agricultural University	Jilin, China
13	Pg_JL2	Jilin Agricultural University	Jilin, China
14	Pg_SC	Sichuan Academy of Agricultural Sciences	Sichuan, China
15	Pg_KS	Kunshan Pengda edible fungus	Shanghai, China

**Table 2:** Methods for determining quantitative characteristics of fruiting bodies.

Code	Characteristics	Method
C1	Individual fruiting body weight/g	Fruiting bodies were picked when pilei were funnel-shaped with a flat edge, and then the weight of an individual fruiting body was measured
C2	Pileus weight/g	Each pileus was separated from its junction with stipe and then weighed
C3	Pileus diameter/mm	The diameter was measured as shown in Figure 1 (3)
C4	Pileus thickness/mm	The thickness was measured as shown in Figure 1 (4)
C5	Gill width/mm	The width was measured as shown Figure 1 (5)
C6	Stipe length/mm	The length was measured as shown Figure 1 (6)
C7	Stipe thickness/mm	The thickness was measured as shown in Figure 1 (7)

## Data Availability

The datasets used and analyzed during the current study are available from the corresponding author upon reasonable request.
